# Effect of Air Pollution on Menstrual Cycle Length—A Prognostic Factor of Women’s Reproductive Health

**DOI:** 10.3390/ijerph14070816

**Published:** 2017-07-20

**Authors:** Anna Merklinger-Gruchala, Grazyna Jasienska, Maria Kapiszewska

**Affiliations:** 1 Faculty of Medicine and Health Sciences, Andrzej Frycz Modrzewski Krakow University, 30-705 Krakow, Poland; maria.kapiszewska@gmail.com; 2 Department of Environmental Health, Faculty of Health Sciences, Jagiellonian University Medical College, 31-531 Krakow, Poland; jasienska@post.harvard.edu

**Keywords:** air pollution, particulate matter, sulfur dioxide, carbon monoxide, nitrogen oxides, menstrual cycle, luteal phase, reproductive health

## Abstract

Air pollution can influence women’s reproductive health, specifically menstrual cycle characteristics, oocyte quality, and risk of miscarriage. The aim of the study was to assess whether air pollution can affect the length of the overall menstrual cycle and the length of its phases (follicular and luteal). Municipal ecological monitoring data was used to assess the air pollution exposure during the monitored menstrual cycle of each of 133 woman of reproductive age. Principal component analyses were used to group pollutants (PM_10_, SO_2_, CO, and NO_x_) to represent a source-related mixture. PM_10_ and SO_2_ assessed separately negatively affected the length of the luteal phase after standardization (b = −0.02; *p* = 0.03; b = −0.06; *p* = 0.02, respectively). Representing a fossil fuel combustion emission, they were also associated with luteal phase shortening (b = −0.32; *p* = 0.02). These pollutants did not affect the follicular phase length and overall cycle length, neither in single- nor in multi-pollutant models. CO and NO_x_ assessed either separately or together as a traffic emission were not associated with overall cycle length or the length of cycle phases. Luteal phase shortening, a possible manifestation of luteal phase deficiency, can result from fossil fuel combustion. This suggests that air pollution may contribute to fertility problems in women.

## 1. Introduction

Ambient air pollution is one of the greatest environmental pressures affecting human wellbeing. The harmful effects of ambient air pollution on many aspects of human health, including the respiratory tract and cardiovascular system is well established [1]. However, despite air quality regulation at a national and trans-national level and despite progress to eliminate the emission of pollutants, urban air pollution continues to rise at an alarming rate [2].

Female fertility and reproductive health respond in a unique way to toxic exposure, especially to those with estrogenic potential [3]. The uniqueness is caused mainly due to mimicking natural hormones activity, and varying regulation and function of the endocrine system [4]. Moreover, effect on reproduction and development are often the result of short-term exposure during the vulnerable periods of ovulation or fetal organogenesis. Adverse effects of some toxicants may not become apparent for years because they accumulate in parental tissue and may be released many years later during pregnancy, lactation, or even post-natal development [5]. In our previous study, we showed that exposure of pregnant women to particulate matter less than 10 μm in size is associated with lower birth weight, and the association is strongest when exposure takes place during the first trimester of pregnancy [6]. These results are in accordance with findings which show the association between the exposure to certain compounds present in ambient air pollution and negative birth outcomes, including fetal growth, neonatal birth weight, pregnancy duration, and infant mortality [7,8,9,10,11]. The knowledge about mechanisms of these phenomena is limited [12,13,14,15]. It is possible that some compounds found in particle matter, especially polycyclic aromatic hydrocarbons which mimic estrogens, may cause the disturbances in hormonal function of the female reproductive system [16,17].

Women’s reproductive physiology, including hormonal function, is reflected by menstrual cycle pattern. Disturbances in any of the stages of menstrual cycle (i.e., follicular and luteal phase) may affect the oocyte quality, ovulation, conception, implantation, or survival of the embryo [18,19]. Thus, the menstrual cycle length and the length of follicular and luteal phases are good prognostic factors of reproductive health. The association between the exposure to environmental pollution and the length of the menstrual cycle, its irregularity, oocyte quality, or miscarriage has received considerable attention in recent years [20,21,22,23].

Although the effects of air pollution on women’s reproductive health and perinatal outcomes have been extensively investigated, the predominant number of published epidemiological studies has assessed single-pollutant exposure [24]. Only a few studies have evaluated the combined effects of environmental exposure on reproductive health [25] and none so far have addressed menstrual cycle characteristics. Identifying environmental factors affecting menstrual cycle and phase-length is vital from the perspective of public health, because it may have important implications for population health, namely infertility, reproductive cancers [26,27], osteoporosis [28], and metabolic disorders [29,30].

Our research was aimed to verify whether the menstrual characteristics of women living in a city with high pollution, specifically the length of the overall menstrual cycle and the length of each of the two phases of the cycle (follicular and luteal), are influenced by the exposure to ambient air pollution. Following the recommendation of the U.S. National Research Council [31], we decided to apply the multi-pollution approach to quantify the air pollution mixture as a whole. We used principal component analyses (PCA), a multivariate statistical technique, to group multiple pollutants such as PM_10_ (particulate matter less than 10 μm), SO_2_ (sulfur dioxide), CO (carbon monoxide), and NO_x_ (nitrogen oxides), to represent a source-related mixture. Several previous studies and reports showed that NO_x_ and CO concentrations can be highly correlated in space and time, indicating their common source as the motor vehicle traffic emissions, whilst correlation of SO_2_ and PM_10_ levels can point to their origin as a stationary fossil fuel combustion [32,33,34,35,36,37]. Moreover, to explore the association between emission sources and menstrual cycle characteristics, we additionally assessed the effect of single pollutants. This approach allows us to determine which components of the emission sources are the most critical in affecting women’s reproductive physiology.

## 2. Materials and Methods

### 2.1. Study Participants

136 Polish urban women from Krakow, Poland, between 24 and 35 years of age were recruited by advertisements between June 2001 and June 2003. They were accepted for participation in the study if they had regular menstrual cycles, no fertility, gynecological and chronic disorders (i.e., diabetes, hypo/hyperthyroidism), did not take any hormonal medication or use hormonal contraception, and had not been pregnant or lactating during the 6 months before recruitment. From the study group, 3 participants were excluded due to following reasons: the lack of date of the beginning of the menstrual cycle and thus impossibility of assessing the pollution exposure (*n* = 1) and very long menstrual cycle, i.e., above 40 days (*n* = 2). Data of 133 participants were used in the analyses. All subjects gave their informed consent for inclusion before they participated in the study. The study was conducted in accordance with the Declaration of Helsinki, and the protocol was approved by the Ethics Committee of Jagiellonian University Medical College (decision number: KTEB/107/B/2000, project identification code: 6 P05D 112 20).

### 2.2. Menstrual Cycle Characteristics

Each woman was asked to keep a diary in which the onset of the monitored menstrual cycle and the onset of the subsequent menstrual cycle were reported. Overall cycle length was calculated as the number of days from menstrual onset to subsequent menstrual onset. During one entire menstrual cycle, each woman collected daily morning saliva samples in order to assess the levels of 17-β estradiol (E2) and progesterone (P). Saliva samples were collected by participants themselves by using sugarless, laboratory tested chewing gum to stimulate salivation into plastic tubes pretreated with a preservative (sodium azide). Samples were stored at ambient temperature during the monitored menstrual cycle. After that time, they were stored in a refrigerator until the date of shipment to the Laboratory of Reproductive Ecology, Harvard University, where samples were frozen at −80 °C until assayed. The collection and storage protocols had been validated in earlier studies [38,39]. Saliva samples of each cycle were analyzed for the concentration of E2 using an I-125-based radioimmunoassay kit (#39100, Diagnostic Systems Laboratories, Webster, TX, USA) with published modifications to the manufacturer’s protocol [40]. P concentrations were analyzed in saliva samples from the last 14 days (reverse cycle day −1 to −14) of each menstrual cycle. P levels were assayed using an I-125 based radioimmunoassay kit (#3400, Diagnostic Systems Laboratories) with published modifications to the manufacturer’s protocol [41].

After completion of assays, the daily concentrations of E2 across each cycle were examined in order to identify the day of the largest midcycle drop in E2 levels which indicates the day of ovulation. For 123 women, the largest midcycle E2 drop was identifiable. The length of the follicular phase was calculated as the number of days between the menstrual onset to the midcycle E2 drop day. The length of the luteal phase was calculated as the number of days between the day after the midcycle E2 drop day to the onset of the subsequent self-reported menses.

### 2.3. Air Pollution Exposure

Krakow, one of the largest city in Poland, faces a serious problem with air pollution. According to a WHO study, Krakow was reported among the principal metropolises with the highest number of days with high PM_10_ concentration [42]. Krakow city is experiencing continuous urban growth, but still has many districts with poor heating systems based on domestic boilers, which are often outdated and inefficient. Moreover, pollution from Silesia, an industrial region of Poland, contributes to the fact that Krakow city, located in the valley, is strongly affected by local and regional atmospheric pollution. Apart from industrial emissions and fossil fuels combustion practices, traffic contributes to high amounts of air pollution in Krakow, like in many other urban areas. All of these factors contribute to rating Krakow amongst the most polluted cities in the world. Poland is among the European countries that contributes the most (i.e., more than 10 percent) to the atmospheric emissions of several key pollutants, including sulfur oxides, particulate matters, and carbon monoxide [43].

The exposure to ambient air pollution during the monitored menstrual cycle of each woman was assessed based on municipal ecological monitoring data. The data for particles with an aerodynamic diameter of ≤10 μm PM_10_ (μg/m³), SO_2_, CO, NOx between 1 January 2000 and 31 December 2003, were obtained from State Environmental Monitoring, the system maintained by the Voivodship Inspectorate for Environmental Protection in Krakow. It provides automatic, continuous measurement of air pollutants, such as PM_10_, SO_2_, CO, NOx, which were measured simultaneously. During the study period, i.e., years: 2000 and 2002–2003 the levels of PM_10_, NOx, and SO_2_ were monitored by 4–5 stations, whilst CO levels by 3 stations within the Krakow city area. This area has the size of 32,685 ha and almost 760,000 inhabitants [44].

The quality of estimates provided are assessed to be high, because of both the number of monitoring stations per city area and location of monitoring stations, specifically: “central” station situated in the Krakow’s Main Square, “traffic” station located on a busy street, representing the traffic zone, “industrial” station located in Nowa Huta eastern district, characterizing both suburban and industrial zones, “urban” station, representing the northern urban background site, and “suburban” station, representing southern urban background site. The air pollution data was available for 82% (PM_10_), 92% (CO), 78% (NO_x_), and 89% (SO_2_) hourly measurements registered by all working stations that monitored pollutants during the study period. The hourly measurements of PM_10_, SO_2_, CO, and NO_x_ were averaged arithmetically across all monitoring stations and 24 h average between the 6 a.m. of a given day to the 7 a.m. the day before was calculated. The reason for this calculation of exposure was to assess the air pollution levels just before the taking of the morning saliva sample by each woman.

### 2.4. Other Measurements of Participants

Body weight measurements were taken twice—before and after monitored menstrual cycle. A detailed description of anthropometric methods and methods of assessment of physical activity was published previously [45,46]. Each woman was asked to fill in seven 24-h precoded food diaries during selected days of her monitored menstrual cycle, representing each day of a week (4 during the follicular and 3 during luteal phase of the cycle). The amount of coffee, tea, and cola drinks were recorded and then the average caffeine intake (mg/day) was assessed based on results of an analytical study on products available on Polish market [47]. The amount of various types of alcoholic drinks, such as beer, wine, vodka, or liquors were also recorded in food diaries to assess the average alcohol intake (g/day) using the Dieta 2 (version 1.1.) computer software (Food and Nutrition Institute, Warsaw, Poland).

Information on birth date, birth weight and length, education (number of years), reproductive history (number of children, age at menarche, usual cycle length), past use of hormonal medication, actual smoking status, and number of cigarettes smoked per day was collected by a questionnaire (partly administered by an interviewer and partly filled by participants). The ponderal index, an indicator of fatness at birth, previously reported to predict menstrual cycle characteristics [48], was calculated as birth weight/(birth length)^3^ and expressed in kg/m^3^.

### 2.5. Statistical Analyses

Testing for potential confounders, we assess the effect of variables that may affect the menstrual cycle length. Pearson’s correlation test was used to verify if any of the continuous variables correlate with menstrual cycle length. The association between any categorical variable and cycle length were examined with one-way analyses of variance (ANOVA). Non-normally distributed variables were log transformed prior to analyses before entering the models.

To assess the joint effect of air pollutants we used Principal Component Analysis (PCA), a statistical technique that transforms a set of inter-related original variables into a set of uncorrelated new variables, the so-called principal components (PCs). PCs are linear combinations of the original variables (pollutants) and are obtained in such a way that the first PC explains the largest fraction of the variance within the original data [49]. We applied Varimax rotation to obtain the rotated factor loadings that represent the contribution of each variable to a specific PC in order to better clarify the influence of each original variable in the PCs. As suggested by the Kaiser criterion, PCs with eigenvalues >1 were retained. The labeling of the PCs retained were based on the highest rotated factor loadings. The mean levels of NO_x_, SO_2_, CO, PM_10_ that characterized women’s exposure during the entire menstrual cycle were included in the PCA as original variables. PCA was also applied for retaining PCs characterized emission sources of pollutants in follicular and luteal phase of menstrual cycles separately. The PCA produced two principal components that explained 80% of the total variance, 95% and 90%, depending on menstrual cycle phases (overall cycle, follicular, and luteal phase, respectively).

The second stage of main analyses was focused on the assessment of the effect of each PCs on menstrual cycle length before and after controlling for confounders. These analyses were done with simple and multiple linear regression models. The same procedure was repeated to assess the effect of joint pollutant exposure on length of follicular and luteal phase of menstrual cycle separately. Standardized (Beta), unstandardized (b), and partial (partial r) regression coefficients were reported. The partial regression plot was used for visualization of the main hypothesis from the multiple regression model: the plot shows the residuals of Y (luteal phase length) on the remaining explanatory variables (age, menarcheal age, alcohol and caffeine intake, smoking status, usual menstrual cycle length, and PC1 scores) vs. residuals of the target explanatory variable (PC2 scores) on the same remaining explanatory variables. This plot accurately reflects the scatter of partial correlation [50].

Following the assessment of the joint effect of exposure, we tested the separate effect of each pollutant, such as NO_x_, SO_2_, CO, PM_10_ on overall menstrual cycle length before and after accounting for confounders. In order to do so, simple and multiple linear regression models were applied. The same procedure was repeated for follicular and luteal phase lengths separately.

The standardized regression (Beta) coefficients were used to compare the effects of single pollutant and PCs of multiple regression models, because Beta coefficients are expressed in units of standard deviations and can be successfully used to make comparisons between models [51]. The significance level was set at 0.05. A *p*-value of less than 0.05 was considered as statistically significant. All the analyses were performed using the STATISTICA package (version 10.0, StatSoft). The 95% CI for the b coefficients was done using the online calculator [52].

## 3. Results

Mean concentrations of PM_10_, SO_2_, NO_x_, and CO that characterized exposure levels during participants’ overall menstrual cycles and menstrual cycle phases are presented on Table 1.

General characteristics of women (*n* = 133) are shown in Table 2. Mean length of the monitored cycles was 28.8 days (SD = 3.74). The average length of monitored follicular phases equaled 15.5 days (SD = 4.06), whilst luteal phases 13.4 days (SD = 1.45). Age of participants ranged from 24 to 35 years. Half of participants had their birth weight higher than 3400 g. Mean ponderal index equaled 22.1 (SD = 6.6). Mean age at menarche was 13.3 years (SD = 1.4). The usual cycle length was no shorter than 29 days among 50% participants with a range between 24–38 days. 38% women in the study (*n* = 50) reported to have at least one child, and the number of children among parous subjects ranged between 1–3. Mean alcohol intake during the monitored cycle was 7.4 g/day (SD = 10.1), whilst mean caffeine intake equaled 243.4 mg/day (SD = 100.9). The average daily caffeine consumption was 4.1 mg/kg of body weight (SD = 1.9). Median (Me) of physical activity equaled 36 MET/day (Q1–Q3 = 32.2–40.7). Body weight ranged from 42.1 to 84.6 kg (Me = 58.5; Q1–Q3 = 52.9–66.3). The average body height equaled 164.6 cm (SD = 6.0). Half of participants had their mean 17-β-estradiol levels equal to or less than 16.4 pmol/L (Q1–Q3 = 11.3–21.4). The mean progesterone levels in the luteal phase was equal to or less than 128.7 pmol/L (Q1–Q3 = 87.6–178.0) among 50% of women. 19.3% of women (*n* = 25) classified themselves as a tobacco smoker at the time of the study. 62.6% of the participants reported to be ever married. The mean duration of education was 16.6 years (SD = 2.7).

None of the characteristics of women were significantly associated with the cycle length, except for age (r = −0.27; *p* < 0.01) and usual cycle length (r = 0.61; *p* < 0.01). Therefore, we decided to include these variables into the final linear regression models as confounders. Additionally, the final models included also smoking status, menarcheal age, and caffeine and alcohol intake because these characteristics have been reported to influence menstrual cycle length [53,54,55].

### 3.1. Effect of Multiple-Pollutant Exposure

Two factors from the principal component analysis characterized the emission sources of pollutants measured throughout the entire menstrual cycle. Factor loadings for those two extracted components after rotation are presented in Table 3. The first PC was positively associated with CO and NO_X_, suggesting motor vehicle emissions; whilst the second had the strong positive loadings on SO_2_ and PM_10_, and might be attributed to emissions from industry and domestic fossil fuel combustion. Similar principal components were extracted for exposure during follicular and luteal phase of menstrual cycle (Table 3).

**Figure 1:**
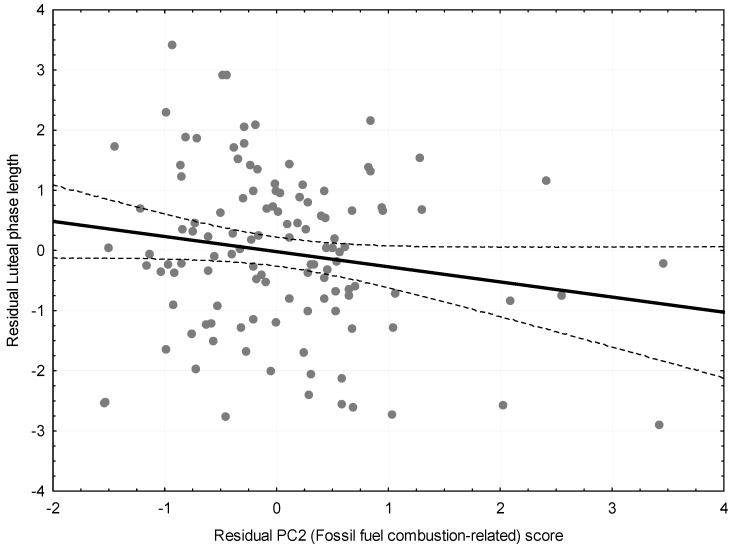
Partial regression plot for the multiple linear regression model reflecting the partial correlation coefficient between Fossil fuel combustion-related air pollutant scores (PC2) and luteal phase length after adjustment for age, menarcheal age, alcohol and caffeine intake, smoking status, usual menstrual cycle length, and PC1. Traffic-related air pollutant scores (r = −0.22, *p* = 0.02). To facilitate the visual assessment of the scatter of points around the line, the 95% confidence intervals were shown. The solid line represents the regression line and the dotted lines stand for the regression lines’ 95% confidence intervals.

The ambient concentrations of two PCs that characterized overall exposure did not affect length of the menstrual cycle, or length of follicular phase, neither before nor after adjustment for potential confounders (Table 4). However, the combined effect of PM_10_ and SO_2_ (PC2) negatively affected the length of luteal phase after standardization for woman’s age, menarcheal age, alcohol and caffeine consumption, smoking status, usual menstrual cycle length, and PC1 scores. Specifically, the regression slope indicated that when age, menarcheal age, alcohol and caffeine intake, smoking status, usual menstrual cycle length, and PC1 factor scores are held constant, an increase of one SD on a PC2 score resulted in a decrease of luteal phase length by 0.32 days (b = −0.32; *p* = 0.02). The increasing PC1 scores were also associated with shortening of the luteal phase length, but this association did not reach the significance level after adjustment for confounders (b = −0.20; *p* = 0.14). The partial regression plot for the multiple linear regression model reflecting the partial correlation coefficient between fossil fuel combustion-related air pollutant scores (PC2) and luteal phase length after adjustment for confounders is shown in Figure 1. The results of the sensitivity analyses further adjusting for the levels of E2 and P were similar to our main analyses (data not shown).

### 3.2. Effect of Single-Pollutant Exposure

The results of single-pollutant models shown that PM_10_, SO_2_, CO, or NOx were not associated with overall cycle length, neither before nor after standardization for age, menarcheal age, alcohol and caffeine intake, smoking status, and usual menstrual cycle length (Table 5). 

There was also no association between any air pollutant and follicular phase length. However, the PM_10_ levels negatively correlated with the luteal phase length after adjustment for confounders. The length of the luteal phase was shortened by 0.02 day per each 1 µg/m^3^ increase in PM_10_ levels during this phase (b = −0.02; *p* = 0.03). SO_2_ exposure also diminished the luteal phase length. The length of the luteal phase was shortened by 0.1 day per each 1 µg/m^3^ increase in SO_2_ levels during this phase (b = −0.06; *p* = 0.02). A similar trend was noted for the association between the levels of CO and luteal phase length, but of borderline significance (b = −0.52; *p* = 0.06). Negative association between ambient concentrations of NOx measured during the luteal phase and the length of this cycle phase did not reach the significance level (b = −0.01; *p* = 0.11).

In order to compare the effects of a joint exposure (emission sources) with the effects of its components (single pollutants), we used standardized slope estimates (Beta coefficients). The standardized effect sizes for PM_10_ and SO_2_ assessed in adjusted single-pollutant models were similar in magnitude to the effect of PC2 on overall menstrual cycle length and the length of each menstrual cycle phase. Similarly, the effects for CO and NO_X_ estimated in adjusted single-pollutant models were also similar in magnitude to the effect of PC1 on overall menstrual cycle length and the length of each menstrual cycle phase.

## 4. Discussion

In this study, we investigated the relationship between the exposure to air pollution experienced by women and their reproductive health reflected by changes in menstrual cycle length. The study participants included healthy women in reproductive age (between 24 to 35 years) with regular menstrual cycles usually ranged between 24 to 38 days long. The concentration of pollutants that women were exposed to were similar in magnitude to PM_10_ levels found in another study conducted in Brazil reporting negative reproductive outcomes [56], but was almost 3 times lower in comparison to average levels of exposure to PM_10_ found in northeastern USA that affected the live birth rates [57]. The mean exposure levels of PM_10_ in our study were also 10 times lower in comparison to the levels of PM_10_ from biomass burning during the cooking hours measured indoors [22]. With regard to the CO and SO_2_ levels, the mean exposure of our participants was almost 3.5–4 times lower (respectively) in comparison to populations’ exposure in other studies that found statistically significant effects on reproductive outcomes [56,58].

Because humans breathe many pollutants simultaneously, and its joint effect on human health may differ from the sum of individual effects of mixtures’ components, we decided to use PCA analyses to assess the combined women’s exposure to pollution. This statistical technique deals with collinearity between pollutants and enables one to derive orthogonal principal components which are not associated with each other. This multipollutant approach follows the recommendations of the U.S. National Research Council [31].

As a result of PCA analyses, the patterns of pollutants were grouped into two classes: traffic-related (PC1, with high loadings on CO and NO_x_) and fossil fuel-related (PC2, with high loadings on PM_10_ and SO_2_). Because PM is also viewed as a marker of road transport emission [59], the weak loadings of PM_10_ on the PC1 were quite surprising. This finding indicates the very weak linear association between ambient PM_10_ levels and concentration of pollutants released from the traffic emission source, such as CO and NO_x_. However, PM_10_ is present in the atmosphere not only due to emissions from the road vehicles but also because due to fossil fuel combustion [36,60]. In Poland, the main sources of total suspended particles emissions are combustion processes [37]. The means of transport and machines with combustion engines have only a 22% share (in 2014) in the total national emission of total suspended particles [37], and perhaps, therefore, we did not see the high loadings of PM_10_ on the PC1. Moreover, CO/NO_x_ and PM_10_/SO_2_ have been confirmed by previous studies and reports to be indicators of traffic emission and fossil fuel emission, respectively [32,33,34,35,36,37]. Stationary combustion of fuels in residential plants and public electricity and heat production are the major sources of emissions of SO_2_ and PM_10_ in Poland, whilst CO and NO_x_ are present among pollutants for which raised concentrations are detected alongside busy roads [37]. The area of Krakow city in Poland, with its coal-based energy has emission sources typical for many metropolitan areas in the new EU member states and countries like China and India [61]. For example, in Taiwanese studies, the identified principal components are similar to those found in our study [32,33].

We found that the combined effect of CO and NO_x_ (i.e., PC1) and the combined effect of PM_10_ and SO_2_ (i.e., PC2) was confirmed by the separate effects seen in single-pollutant models after adjustment for woman’s age, menarcheal age, alcohol and caffeine consumption, smoking status, and usual menstrual cycle length. Air pollutants such as PM_10_ and SO_2_ assessed (separately) one by one negatively affect the length of the luteal phase after standardization to confounders (i.e., woman’s age, menarcheal age, alcohol and caffeine consumption, smoking status, and usual menstrual cycle length). When analyzed as indicators of common emission sources of fossil fuel combustion, they were also associated with luteal phase shortening. They did not affect the follicular phase length and overall cycle length, neither in a single-pollutant nor in multi-pollutant models. Other pollutants such as CO and NO_x_ assessed either separately or together as a traffic emission source were not associated with overall cycle length or the length of menstrual cycle phases.

The association between menstrual cycle characteristics and single, but not multi-pollutant exposure, has been shown also by others, however the amount of evidence is very limited. Indoor exposure to PM_10_ from biomass burning has been associated with 5 times increased risk of shortened menstrual cycle [22]. Cigarette smoking, both current and ex-smoking, an exposure to many toxic substances that are found also in PM_10_, such as polycyclic aromatic hydrocarbons or metals, correlated with shorter and irregular cycles [62,63], shorter follicular and luteal phase lengths [63,64], and duration of dysmenorrhea [65]. Our findings support many studies that reported association between exposure to ambient air pollution and adverse reproductive and pregnancy outcomes, including miscarriage, birth defects, impaired intrauterine growth, low birth weight, and premature birth [22,66,67]. The successful human pregnancy and delivering of a healthy infant depends in many ways on having regular menstrual cycles and adequate hormone production [68]. Negative effects of exposure to pollution on reproductive health strongly indicate the disturbances in hormonal function which, in turn, are expressed in changes in menstrual cycle length. Thus, the length of the stages of menstrual cycle, such as the follicular and luteal phases, also reflects the levels of estrogens and progesterone secretion [69,70]. Air pollutants, especially those which are recognized as xenoestrogens, can be responsible for those disturbances [17].

The majority of the compounds which constitute the PM are xenoestrogens. Among the chemicals absorbed from the PM, polycyclic aromatic hydrocarbons (PAHs) have been implicated in the etiology of adverse reproductive function, including reduction or destruction of follicles and infertility, or reproductive disorders, such as breast cancer [71]. The concentration of PAHs in the PM sample is considered an index of biologically active compounds [72]. PAHs and their metabolites may exhibit estrogenic activity [73] due to their ability to activate the Ah receptor involved in xenobiotic metabolism. Because of that, they are able to bind and activate the estrogen receptors in target tissues which can result in changes in the metabolism of reproductive hormones [74].

The changes in the luteal phase of the menstrual cycle, especially shortening of its length, may be characterized by disturbances of the growth of a new thickened lining of the uterus, named the endometrium, which is crucial for implantation of a fertilized egg [75]. An adequately prepared endometrium is necessary for the development of a healthy pregnancy. Luteal phase deficiency, although described in healthy normally menstruating women, may preclude embryo implantation and induce spontaneous miscarriage [75].

There is some evidence that air environmental factors may affect the conception and implantation processes and increase miscarriage risk. A higher incidence of implantation failures was seen in a group of mice exposed to elevated PM_10_ and NO_2_ levels compared with a filtered air group [76]. Exposure to ambient PM may also disrupt the lineage specification at the blastocyst stage without interfering in early development of the mouse embryo [77]. Reproductive failures were also noted among human populations. High levels of PM_10_ exposure during the follicular phase of the conception cycle was associated with a 2.6-fold increase in the risk of miscarriage in couples undergoing in vitro fertilization and embryo transfer [23]. Likewise, increased NO_2_ levels among a similar group of women was associated with lower live birth rates [57]. Higher risk of spontaneous abortions and infertility was noted also among female traders working in dusty environments in the center of Durban city in South Africa, who were also exposed to biomass fuel smoke [20]. An increased risk of fetal loss in early pregnancy was also noted after exposure to high levels of SO and total suspended particles during the first month of pregnancy [78]. Furthermore, high exposure to SO_2_, especially during the second month before conception, may also negatively affect the probability of conception. An inverse association between the concentration of SO_2_ during that time and conception success in the first unprotected menstrual cycle was observed in women living in a highly polluted area in the Teplice region in the Czech Republic [58].

The success of conception depends also on oocyte quality. The length of the menstrual cycle and luteal phase was shown to be reliable indicators of oocyte quality and ovarian reserve [79]. Longer menstrual cycles are related to improved embryo quality and higher pregnancy and delivery rates in assisted reproduction [19]. A menstrual cycle of 34–35 days correlated well with higher oocyte quality and better response to ovarian stimulation in comparison to shorter cycles [80]. On the other hand, short luteal phases have been reported to be associated with infertility and habitual spontaneous abortions [80]. Therefore, overall cycle length and menstrual cycle phase length can be treated as easy-to-measure indicators (biomarkers) of women’s fertility, independently of other menstrual cycle characteristics. Length of menstrual cycle and its phases can be independent from other characteristics of the cycle (e.g., ovarian hormone and gonadotropin levels) among healthy women of reproductive age [81]. Moreover, examining the multiple components of the menstrual cycle, Barrett et al. (2013) revealed in principal component analysis that overall cycle length together with follicular phase length loaded on a different factor than luteal phase length, which suggested that these factors are independent of one another. Further, luteal phase length is considered to be more constant across multiple cycles of a woman, in contrast to overall cycle and follicular phase length that are more variable from cycle to cycle [82]. This observation supports the idea that luteal phase length is an independent factor.

Our study, just like all studies based on municipal ecological monitoring data, is not free from several limitations. One of them is potential misclassification of exposure. Women living in metropolitan locations are not exposed to pollutants only in their place of residence, but also in their workplace, during their travel to work, and all other activities, such as shopping, recreational activities, etc. Therefore, their exposure might be better represented by the average, rather than by just one station closest to the home address. This issue poses a real challenge which has also been discussed by others [56]. Assessing the exposure by averaging the levels of pollution from several monitors and not by assigning the air pollution data from the closest monitor may be connected with error, but it is likely to be non-differential. Moreover, the most serious consequence of such an error is the attenuation of the effect estimated [83]. Moreover, even when considering the estimation of exposure for populations living nearby the monitoring station in epidemiological sites, a distance of 50 km is very often chosen, as a reasonable distance for extrapolation of observed air pollutant concentrations [84,85,86,87]. Whilst the Krakow city covers an area of 18 km × 31 km which, during the study period, it was scattered with 3–5 monitoring stations. Therefore, it seems rational to assume that the estimation of exposure in our study population is reliable. Additionally, after taking into account the spatial resolution assessment based on regulatory monitoring network data of other study sites, we noted that the number of monitoring stations per land area in Krakow is high (almost 6/300 km^2^) in comparison to locations in other studies, for example California’s South-Cost Air Basin (0.6/300 km^2^) and even Vancouver (1.5/300 km^2^), which was considered to have very high monitoring density [88]. The high quality of the network of air pollution stations in the Malopolska province and its appropriate coverage of the Krakow residency area (3–5 stations per 32,685 ha in the city of almost 760,000 inhabitants [44]) was also confirmed by a cohort study conducted on women of reproductive age who were residents of the city of Krakow [89]. Additionally, with regard to exposure assessment, we are aware that taking into account only four of all pollutants, namely CO, NO_x_, PM_10_, and SO_2,_ makes it likely that only a part of the total pollutant mix is captured. This procedure was motivated by an unavailability of data on the one hand, and commonly followed practice on the other hand [90].

Another limitation of our study is not taking into account the variation of cycle and phase length over a long period of time. Monitoring the length of the only one menstrual cycle per women may have been imprecise to capture this variation. However, our participant’s cycle length and cycle phases length were typical of healthy women in this age [81,91]. Further research is needed to determine whether these results also apply to younger and older women, whose cycles’ durations may be less typical.

Shortening of the luteal phase length as a result of pollution exposure may have clinical implications, especially when considering the fact that women are constantly and inevitably affected by harmful toxicants from inhaled air. According to the opinion of the Practice Committee of the American Society for Reproductive Medicine [18], if luteal phase deficiency is constantly present in many cycles of the same women, it can be considered clinically relevant. However, our findings of the reduction of 0.3 days in the luteal phase length after increasing by 1 standard deviation exposure to fossil fuel-related pollutants, due to its weak effect, should be interpreted with caution. More studies in other settings needs to be conducted to confirm our results. Further research aimed at investigating relationships between air pollution and female reproductive physiology is also warranted to explore mechanisms involved in these phenomena. In particular, it would be interesting to analyze the impact of air pollutants on women’s reproductive hormone concentrations.

## 5. Conclusions

The air pollutants PM_10_ and SO_2_, assessed separately, negatively affect the length of the luteal phase after standardization to confounders, such as woman’s age, menarcheal age, alcohol and caffeine consumption, smoking status, and usual menstrual cycle length. As markers of fossil fuel combustion emission sources, these pollutants were also associated with statistically significant luteal phase shortening. Exposure to pollutants did not affect the follicular phase length and overall cycle length, neither in single- nor in multi-pollutant models. Other evaluated pollutants, such as CO and NO_x_, assessed either separately or together as a traffic emission source, were not associated with overall cycle length or the length of menstrual cycle phases. Luteal phase shortening, a possible manifestation of luteal phase deficiency, can be affected by air pollution, specifically by the toxicants released by fossil fuel combustion.

## Figures and Tables

**Table 1:** Mean air pollutants concentrations (PM_10_, SO_2_, NO_x_, and CO) characterized the exposure levels during participants’ menstrual cycle and cycle phases during the study period (years 2000–2003).

Pollutant	*n*	Mean (SD)
PM_10_ (µg/m^3^)		
overall cycle	133	61.7 (13.2)
follicular phase	123	66.9 (22.2)
luteal phase	123	55.5 (14.7)
SO_2_ (µg/m^3^)		
overall cycle	133	16.4 (5.4)
follicular phase	123	17.9 (7.3)
luteal phase	123	14.9 (5.5)
CO (mg/m^3^)		
overall cycle	133	1.3 (0.5)
follicular phase	123	1.3 (0.6)
luteal phase	123	1.2 (0.5)
NO_X_ (µg/m^3^)		
overall cycle	133	121.4 (45.7)
follicular phase	123	127.7 (52.0)
luteal phase	123	116.3 (47.3)

SD—standard deviation.

**Table 2:** Characteristics of participants and their association with menstrual cycle length.

Characteristics (Continuous)	*n*	Mean	SD	Pearson r Coeff.	*p*-Value
Age (years) *	132	29.5	3.13	−0.27	<0.01
Age at first child (years)	50	24.1	3.11	0.15	0.30
Birth weight (g) *	112	3306.6	632.76	0.05	0.59
Birth length (cm)	102	53.5	3.96	0.02	0.81
Ponderal index (kg/m^3^)	102	22.1	6.63	0.00	0.98
Menarcheal age (years)	129	13.3	1.40	0.02	0.80
Height (cm)	133	164.6	5.96	−0.02	0.79
BMI (kg/m^2^) *	133	22.2	2.90	−0.07	0.41
Body weight difference (kg)	129	−0.6	1.39	0.04	0.65
Mean body weight (kg) *	132	60.2	8.79	−0.07	0.40
Physical activity (sum of MET/day) *	102	37.9	8.16	0.02	0.88
Education (years)	131	16.6	2.74	0.11	0.20
Caffeine intake (mg/day)	130	243.4	100.93	−0.03	0.76
Alcohol intake (g/day)	130	7.4	10.12	−0.14	0.12
Usual cycle length (days) *	128	29.2	2.61	0.61	<0.01
Mean E2 levels (pmol/L) *	133	17.9	9.78	0.12	0.15
Mean P levels (pmol/L) *	134	136.3	64.24	0.14	0.11
**Characteristics (Categorical)**	***n***	**Mean**	**SD**	**F**	***p*-Value**
Parity					
no	82	29.2	4.10	F(1,130) = 2.92	0.09
yes	50	28.1	2.98		
Marital status					
single	49	29.1	3.67	F(1,129) = 0.65	0.42
ever married	82	28.6	3.81		
Smoking status					
non-smoker	104	28.8	3.54	F(1,127) = 0.03	0.87
smoker	25	28.9	4.57		

* Pearson correlation test with log-transformed variable.

**Table 3:** Factor loadings after Varimax rotation of mean levels of pollutants measured during menstrual cycles (overall menstrual cycle, follicular, and luteal phase).

Pollutant	PC1 Traffic-Related	PC2 Fossil Fuel-Related
Overall cycle		
PM_10_ mean	−0.11	0.76
SO_2_ mean	0.05	0.79
CO mean	0.99	−0.02
NO_x_ mean	0.99	−0.03
Eigenvalue	1.99	1.21
Variance explained	50%	30%
Follicular phase		
PM_10_ mean	0.16	0.94
SO_2_ mean	0.14	0.94
CO mean	0.98	0.16
NO_x_ mean	0.99	0.12
Eigenvalue	1.99	1.81
Variance explained	50%	45%
Luteal phase		
PM_10_ mean	0.19	0.88
SO_2_ mean	0.25	0.86
CO mean	0.96	0.23
NO_x_ mean	0.98	0.16
Eigenvalue	1.99	1.60
Variance explained	50%	40%

Two PCs have eigenvalues of >1 and account for 80% of variance, 95% and 90%, depending on menstrual cycle phases (overall cycle, follicular and luteal phase, respectively); PM_10_: particulate matter; SO_2_: sulfur dioxide; CO: carbon monoxide; NO_x_: nitrogen oxides.

**Table 4:** The association between principal components (PCs) and menstrual overall cycle and phases length tested by simple and multiple regression analyses.

Principal Component (PC)	Crude			Adjusted *			
	Beta Coeff.	b Coeff. (95% CI)	*p*	Beta Coeff.	b Coeff. (95% CI)	Partial r Coeff.	*p*
Overall cycle							
PC1 traffic-related	0.01	0.03 (−0.52 to 0.58)	0.93	0.00	0.01 (−0.54 to 0.56)	0.00	0.98
PC2 fossil fuel-related	−0.09	−0.32 (−0.85 to 0.21)	0.33	0.00	0.00 (−0.53 to 0.53)	0.00	0.99
Follicular phase							
PC1 traffic-related	0.00	0.00 (−0.71 to 0.71)	0.99	0.02	0.07 (−0.56 to 0.70)	0.02	0.83
PC2 fossil fuel-related	−0.10	−0.41 (−1.14 to 0.32)	0.26	−0.03	−0.13 (−0.74 to 0.48)	−0.04	0.66
Luteal phase							
PC1 traffic-related	−0.10	−0.15 (−0.41 to 0.11)	0.27	−0.14	−0.20 (−0.48 to 0.08)	−0.14	0.14
PC2 fossil fuel-related	−0.13	−0.18 (−044 to 0.08)	0.16	−0.22	−0.32 (−0.60 to −0.04)	−0.22	0.02

* adjusted for age, menarcheal age, alcohol and caffeine intake, smoking status, usual menstrual cycle length, and PC other than estimated.

**Table 5:** The association between single air pollutants and menstrual overall cycle and phases length tested by simple and multiple regression analyses.

Pollutant	Crude			Adjusted *			
	Beta Coeff.	b Coeff. (95% CI)	*p*-Value	Beta Coeff.	b Coeff. (95% CI)	Partial r Coeff.	*p*-Value
Overall cycle							
PM_10_ mean	−0.15	−0.04 (−0.08 to 0.00)	0.09	−0.04	−0.01(−0.05 to 0.03)	−0.05	0.56
SO_2_ mean	−1.01	0.00 (−0.12 to 0.12)	0.94	0.04	0.02 (−0.08 to 0.12)	0.05	0.56
CO mean	−1.01	−0.06 (−1.31 to 1.19)	0.93	0.00	−0.01 (−1.10 to 1.08)	0.00	0.99
NO_x_ mean	0.00	0.00 (−0.02 to 0.02)	0.96	0.00	0.00 (−0.02 to 0.02)	0.00	0.99
Follicular phase							
PM_10_ mean	−0.15	−0.03 (−0.07 to 0.01)	0.10	−0.05	−0.01 (−0.03 to 0.01)	−0.07	0.48
SO_2_ mean	−0.05	−0.03 (−0.13 to 0.07)	0.61	−0.01	0.00 (−0.08 to 0.08)	−0.01	0.94
CO mean	−0.02	−0.12 (−1.39 to 1.15)	0.85	0.01	0.05 (−1.06 to 1.16)	0.01	0.92
NO_x_ mean	−0.01	0.00 (−0.02 to 0.02)	0.91	0.08	0.00 (−1.02 to 0.02)	0.02	0.84
Luteal phase							
PM_10_ mean	−0.12	−0.01 (−0.03 to 0.01)	0.17	−0.21	−0.02 (−0.04 to −0.00)	−0.21	0.03
SO_2_ mean	−0.14	−0.04 (−008 to 0.00)	0.12	−0.23	−0.06 (−0.11 to −0.01)	−0.21	0.02
CO mean	−0.13	−0.37 (−086 to 0.12)	0.15	−0.18	−0.52 (−005 to 0.01)	−0.18	0.06
NO_x_ mean	−0.11	0.00 (0.00 to 0.00)	0.21	−0.15	−0.01 (−0.02 to −0.00)	−0.15	0.11

* adjusted for age, menarcheal age, alcohol and caffeine intake, smoking status, and usual menstrual cycle length.
